# Development and Evaluation of a Faculty Teaching Boot Camp Before and During the COVID-19 Pandemic

**DOI:** 10.7759/cureus.26237

**Published:** 2022-06-23

**Authors:** Ben A Blomberg, Fei Chen, Gary L Beck Dallaghan, Judson MacDonald, Lindsay Wilson

**Affiliations:** 1 Department of Medicine, Division of Geriatrics, University of North Carolina at Chapel Hill School of Medicine, Chapel Hill, USA; 2 Department of Anesthesiology, University of North Carolina at Chapel Hill School of Medicine, Chapel Hill, USA; 3 Office of Medical Education, University of North Carolina at Chapel Hill School of Medicine, Chapel Hill, USA; 4 Learning and Development, Les Mills United States, Chapel Hill, USA

**Keywords:** teaching boot camp, online teaching, covid-19, virtual workshop, faculty development in medical education

## Abstract

Introduction

Medical faculty often assume teaching responsibilities without formal training in teaching skills. The purpose of this study was to design, implement, and evaluate boot camp workshop training faculty in basic teaching competencies. We also describe the transition to a virtual format necessitated by the COVID-19 pandemic.

Methods

The workshop content was derived from a needs assessment survey and discussion with content experts. Four main content areas were identified: setting expectations, giving feedback, evaluating learners, and teaching in specific settings (outpatient clinics, inpatient wards, procedures/surgery, and small groups). The initial boot camp was a four-hour in-person event. The following year, the boot camp was offered via online videoconference. We used a pre-post survey to assess participant reaction and knowledge acquisition from session content.

Results

A total of 30 local faculty attended the 2020 in-person boot camp, while 105 faculty from across the state attended the 2021 online boot camp. Statistically significant increases in post-knowledge scores were identified for two sessions in the 2020 boot camp and four sessions in 2021. The participants rated both boot camps favorably with no significant difference between the in-person and online presentations for most ratings but were less satisfied with networking opportunities in the online boot camp.

Discussion

We describe an effective faculty development boot camp teaching core competencies for medical clinician-educators. We were able to leverage the online teleconferencing platform to deliver the content to a larger number of preceptors at distant sites without sacrificing outcomes of participant satisfaction and improvement in knowledge scores. The online model allowed busy clinicians to participate while multitasking. Comments also highlighted the importance of having an engaged moderator during the online event.

Conclusions

Many medical schools utilize preceptors in distant locations. We demonstrated the feasibility of reaching a much larger and geographically widespread group of clinical preceptors using a virtual format while still showing improvement in knowledge scores relating to workshop content. For future faculty development, we propose that hybrid models with both in-person and virtual components will be effective in meeting the needs of a geographically distributed faculty.

## Introduction

Despite their lack of formal training in education, faculty in medicine are expected to teach medical students, residents, and other trainees. To achieve competence in teaching, faculty must acquire knowledge and gain skills, often through ad hoc experiences and observation of skilled educators [1,2].

To address this need for formal training in education, we recognized an opportunity to design a teaching boot camp that would be targeted at junior faculty [3]. This would help foster the early development of teaching competencies among medical faculty and a more uniform experience for learners at our institution.

There are a variety of theories of teaching that could have been considered in the development of the boot camp. We considered the challenges inherent in teaching trainees in a dynamic patient care system. With that in mind, we focused on the Meutic Theory of Teaching, which is often referred to as the Socratic method. This theory assumes that questioning techniques aid in the recollection and transfer of knowledge [4]. Another theory relevant to teaching in the health sciences is the Experiential Learning Theory, which draws heavily on Kolb’s work [5]. This theory highlights the need for different experiences involving observation, concrete examples, abstraction, and active experimentation [6]. With experiential learning, learners take responsibility for their learning, reflecting on experiences to improve their performance.

Srinivasan et al. [7] described a set of core competencies for medical educators adapted from the Accreditation Council for Graduate Medical Education (ACGME) clinical competencies. Informed by these core teaching competencies, we selected four main content areas to cover in our boot camp: setting expectations, giving feedback, evaluating learners, and teaching in specific settings (outpatient clinics, inpatient wards, procedures, small groups, and lectures). These topics aligned with the theories we identified; specifically, setting expectations involves concrete examples leading to experiential learning. Giving feedback involves questioning techniques and abstraction. Evaluating learners requires observation of learners but also takes into consideration questioning techniques. Teaching in specific settings aligns with active experimentation.

Using this framework and an internal needs assessment, we developed a four-hour boot camp targeting core teaching competencies for junior faculty at our medical school, which was implemented in February 2020. Shortly thereafter, the COVID-19 pandemic transformed the way educational content could be delivered across the globe. In this paper, we describe the design, implementation, and evaluation of our initial teaching boot camp and the successful transition of the boot camp into a virtual format.

## Materials and methods

Needs assessment

Using an iterative process of discussion and item review among the authors, we developed a needs assessment survey informed by the teaching competencies proposed by Srinivasan et al. [7], as well as informal discussions with 14 local content experts. These individuals are all University of North Carolina (UNC) School of Medicine faculty members who ultimately also presented sessions as part of the initial teaching boot camp.

The 17-item web-based survey included Likert scales assessing confidence in the ability to give feedback, evaluate learners, set expectations, create a positive learning environment, and teach in various clinical and didactic settings. We also included multiple-choice and free-response items soliciting additional topics for potential inclusion in the boot camp and assessing items of interest, including prior teaching experience and training, current teaching responsibilities, and interest in participating in the planned teaching boot camp. One content expert from within the institutional Academy of Educators (AOE) reviewed the survey items for clarity. We also used informal discussions with local content experts to solicit didactic topics for inclusion in the boot camp.

We distributed the survey in September 2019 using the new faculty and Academy of Educators (AOE) email listservs within our institution. We also solicited survey responses from physicians who serve as community preceptors for medical students at our institution. Recruitment of community preceptors was primarily by word-of-mouth, as the AOE does not maintain a listserv of community preceptors. The survey was kept open for two weeks, and two reminders were included in the weekly AOE newsletter for two weeks. Responses were anonymous, and no incentive was offered for survey completion.

Nineteen faculty and 23 community preceptors responded to the survey. The respondents expressed interest in learning more about giving feedback, interactive small group teaching methods, evaluating students, and strategies to efficiently incorporate students into outpatient clinic workflows. The results of the needs assessment informed our selection of four main content areas to cover in our boot camp: setting expectations, giving feedback, evaluating learners, and teaching in specific settings (outpatient clinics, inpatient wards, procedures, and small groups).

Boot camp content

Feedback

In the in-person boot camp, the feedback session was led by three faculty members and 10 fourth-year medical students. The session began with a Think-Pair-Share [8] to prompt past examples of “good” and “bad” feedback, relative to each participant’s experience. Next, a didactic overview of giving feedback to students was offered, listing characteristics of effective feedback, exploring specific frameworks for phrasing and organizing feedback (Coyle’s Framework for Providing Better Feedback and the Radical Candor Model for Feedback) [9,10], and highlighting emotional triggers that can prevent learners from receiving feedback well [11]. A video was played next, demonstrating a role-play scenario in which a student provided an ineffective patient sign-out at shift change. Following the video, the participants were separated into small groups of 3-4. Each group had one medical student join them. The participants in each small group practiced offering feedback to their medical student on the sign-out observed in the video. Following this practice, the students offered feedback on the feedback they received from each participant. For the virtual boot camp, medical students did not participate due to the inability to recruit enough student volunteers for all of the small groups given the much larger number of participants. Faculty were placed into breakout rooms to role-play giving and receiving feedback. They provided feedback to each other on their feedback techniques.

Setting Expectations and Learner Evaluation

In this joint session, the time was split between two topics: setting expectations with learners (led by one faculty member and one resident) and evaluating learners (led by two faculty members). The session primarily used small group discussion to brainstorm the value in explicitly creating a shared mental model of course expectations, often referred to as a classroom culture [12]. The presenters then outlined key components of setting expectations for every team member with a focus on the inpatient context. Faculty were asked to create their own list of expectations on a card that they would distribute to their learners in the future. Next, the small groups shifted the focus to learner evaluation. The faculty presenters introduced the concepts of formative and summative assessment [13,14] and reviewed the specific evaluation tools in place for students and residents at our institution. The presenters discussed the important components of reliability and validity in assessment, including the role of implicit bias. Examples of effective and ineffective assessments were shared and discussed. Presenters also demonstrated how to effectively navigate evaluation forms utilized by our institution and deliver narrative comments for formative and summative feedback using these forms. For this session, the curriculum was not changed for the virtual boot camp.

Working With Students in Clinic/Wards/Surgery/Small Groups

These breakout sessions were offered to conclude the day. Each group held a focused discussion on strategies to teach effectively in one of the four settings mentioned above. Each group included a brief didactic piece by the faculty presenter with expertise in that setting. This was followed by a group discussion to allow for sharing of experience and brainstorming of teaching strategies. In working with learners in the clinic, the participants discussed efficiently incorporating a learner through schedule modifications and using pre-clinic time for student learning and reflected on high-impact interventions for effective teaching in the clinic such as using direct observation [15]. In working with students on the wards [16], the participants discussed resources to improve bedside teaching and coaching techniques. In working with students in surgery, the participants discussed techniques to help maximize learning and practice in the operating room. In working with small groups, the participants discussed strategies for creating a safe and respectful environment for small group learning, techniques for encouraging student participation in small groups, and methods of addressing more challenging student behavior (e.g., student who dominates the discussion). For these sessions, the curriculum was not changed for the virtual boot camp.

Logistics and delivery

**Figure 1:**
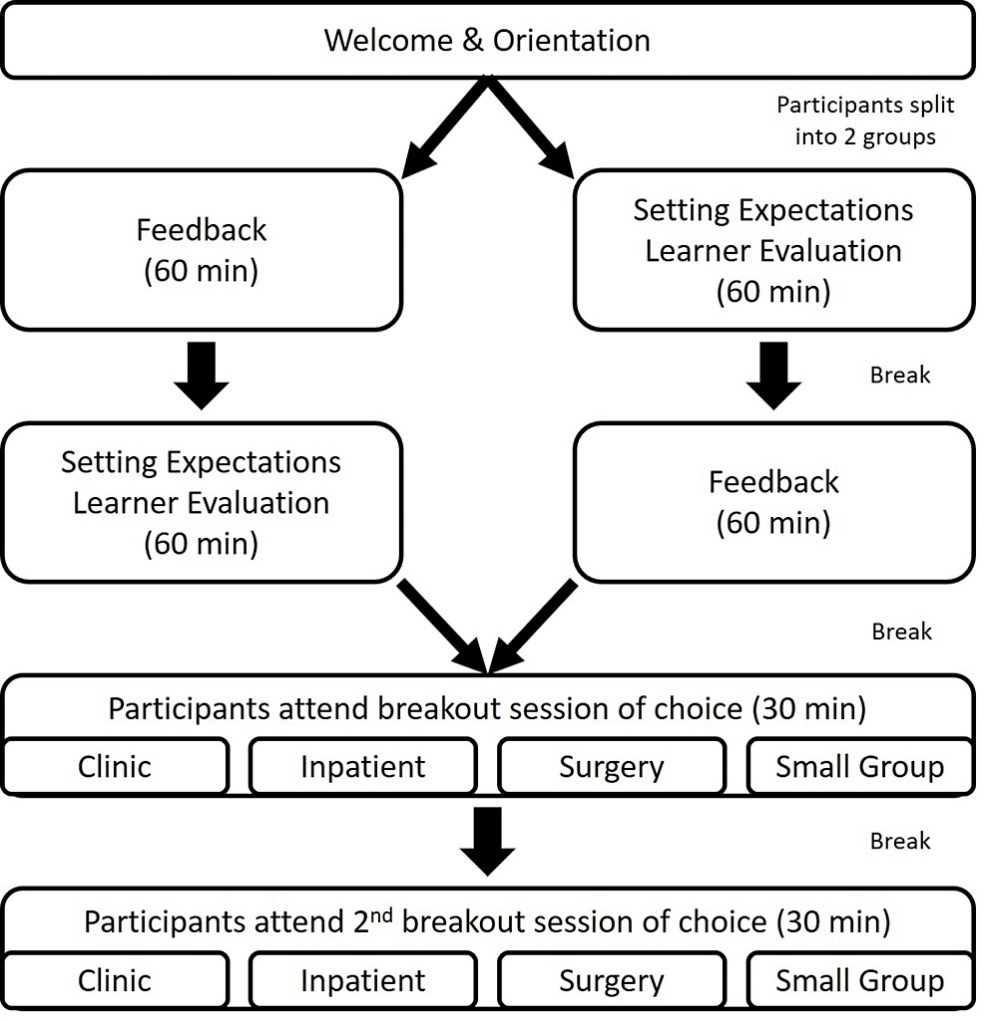
Boot Camp Sessions

For the in-person boot camp, we obtained internal funding for the boot camp, which allowed us to host the event at a local conference center, as well as provide lunch and snacks. We delivered the virtual boot camp via the Zoom online teleconferencing platform and utilized breakout rooms. The event ran for four hours in one afternoon. Figure 1 demonstrates how participants proceeded through the different sessions.

During the online boot camp, the program coordinator utilized a playlist of music during the opening, transition periods, breaks, and closing to maintain energy and excitement [17]. To enhance the engagement of session participants, intentional methods of eliciting responses were used, such as polls, live-tweeting, QR codes to website resources, and verbal encouragement to use the chat function in Zoom to interact with other session participants. The latter was used to also encourage networking among the participants, as the virtual format did not afford the candid moments that participants get when transitioning between sessions, grabbing snacks, or using the restroom. In January 2021, participants were also offered the opportunity to receive CME credit for each session attended.

Boot camp evaluation

To assess knowledge acquisition and attitudes toward the boot camp, we designed a web-based pre-post survey via Qualtrics. The participants generated an anonymous alphanumeric subject identifier based to allow matching of pre-post surveys. Each session facilitator contributed 2-4 multiple-choice questions, which we reviewed and edited for clarity and consistency. Branching logic was used so that the participants only responded to questions for the two breakout sessions they planned to attend. The post-survey contained additional items assessing attitudes toward the overall boot camp. The post-survey remained open for two days after the close of the event. Responses were anonymous, and no incentive was offered for survey completion. For the online boot camp, we added items to the post-survey to assess participants’ attitudes toward the online format.

The normality of distribution was assessed visually using Q-Q plots. The assumption of normal distribution of the differences between the pre- and post-survey pairs of data was not met. Thus, Wilcoxon signed-rank tests were used to compare the pre- and post- differences in knowledge test scores in each year. For the same non-normal distribution reason, Wilcoxon-Mann-Whitney and Fisher’s exact tests were used to compare the difference in perceived experience reported in the post-surveys between the two years. A p-value of less than 0.05 was considered significant for all statistical tests. Statistical analysis was performed using SAS 9.4 (SAS Institute Inc., Cary, NC, USA).

## Results

Table 1 demonstrates the key statistics of both teaching boot camps, pertaining to the participants, their participation in the pre- and post-evaluation survey, and the average length of teaching experience. The participants were almost entirely clinical faculty, with a few basic science faculty participating in the online boot camp.

**Table 1:** Boot Camp Participation

	2020 (In-Person)	2021 (Virtual)
# Registered	41	149
# Attended	30	105
# Participated in Pre-evaluation Survey	28 (93%)	99 (94%)
# Participated in Post-evaluation Survey	25 (83%)	66 (63%)
Average # of Years Teaching	7.8 years	8.5 years

Table 2 demonstrates pre-post knowledge item scores for the two boot camps. There was a trend toward improvement for all sessions in both years. In 2020, statistically significant improvement was noted for two of the six sessions (feedback and setting expectations) included in the analysis. In 2021, statistically significant increases in post-knowledge scores were seen in four of the six sessions (feedback, setting expectations, teaching on the wards, and teaching in small groups). For evaluation scores in both years, a high proportion of sessions have maximum scores on the observed variable. Of note, teaching in clinic items were excluded from the analysis due to being erroneously set up as single-response questions in 2020, which was corrected in 2021. Surgery teaching questions were also excluded from the 2020 analysis because only one participant completed these questions in both pre- and post-surveys in 2020.

**Table 2:** Pre- and Post-Boot Camp Knowledge Scores ^a^The maximum score for each session varied due to different numbers of questions for each session (feedback=4, evaluation=3, set expectation=2, ward=4, small group=3, surgery=2). ^b^Table 2 only included those who completed both pre- and post-surveys. Those who only responded to pre- or post- were not included in the analysis.

2020 (In-Person)					
Session^a^	N^b^	Mean Pre-score	Mean Post-score	Mean Score Improvement	p
Feedback	18	0.889	2.056	1.167	0.0005
Evaluation	18	2.389	2.667	0.278	0.1797
Setting Expectations	18	0.889	1.444	0.556	0.0273
Teaching on the Wards	6	1.833	2.833	1.000	0.2500
Teaching in Small Groups	6	1.000	1.500	0.500	0.5000
2021 (Virtual)					
Feedback	54	1.315	2.167	0.852	<0.0001>
Evaluation	54	2.574	2.704	0.130	0.1185
Setting Expectations	58	1.121	1.603	0.483	<0.0001>
Teaching on the Wards	15	1.667	2.800	1.133	0.0020
Teaching in Small Groups	29	0.655	2.068	1.413	<0.0001>
Teaching in Surgery	13	0.077	0.231	0.154	0.5000

Table 3 demonstrates participant evaluations of the boot camp content in 2020 and 2021. Overall, the participants rated the boot camp favorably, with the majority rating the content “very helpful” or “extremely helpful.” For most ratings, there was no significant difference between boot camps, with the exception that for the virtual boot camp, more participants rated the content as “somewhat helpful.”

**Table 3:** Participant Experience SD: standard deviation ^a^Based on a 5-point Likert scale (1 being unsatisfactory and 5 being extraordinary)

Logistics			
Item^a^	2020 (In-Person): Mean (SD), n=25	2021 (Virtual): Mean (SD), n=66	p
Programming of the Event	4.76 (0.53)	4.64 (0.62)	0.346
Quality of the Content Delivered	4.48 (0.71)	4.55 (0.59)	0.858
Quality of Handouts/Materials	4.28 (0.84)	4.29 (0.86)	0.950
Overall Experience	4.64 (0.57)	4.56 (0.61)	0.587
Content			
Scale	2020 (In-Person)	2021 (Virtual)	p
Extremely Helpful	11 (44%)	29 (43.9%)	0.037
Very Helpful	13 (52%)	26 (39.4%)	
Somewhat Helpful	0 (0%)	11 (16.7%)	
Not So Helpful	1 (4%)	0 (0%)	
Not at All Helpful	0 (0%)	0 (0%)	
Likeliness to Recommend to Others			
Scale	2020 (In-Person)	2021 (Virtual)	p
Very Likely	22 (88%)	53 (80.3%)	0.594
Somewhat Likely	2 (8%)	11 (16.7%)	
Not Likely	1 (4%)	2 (3%)	

Table 4 demonstrates a set of data from only the virtual edition of the boot camp. The majority of the participants reported agreeing that the platform was accessible and easy to navigate, although responses were mixed with regard to the social networking opportunities afforded by the virtual experience.

**Table 4:** 2021 Evaluation of Virtual Format

n=66	To what degree do you agree with the following statements?				
Statement	Strongly Disagree	Somewhat Agree	Neither Agree nor Disagree	Somewhat Agree	Strongly Agree
The virtual event platform was accessible.	2 (3%)	3 (4.6%)	1 (1.5%)	3 (4.6%)	57 (86.4%)
I found ease in navigating the virtual event platform.	2 (3%)	7 (10.6%)	0 (0%)	12 (18.2%)	45 (68.2%)
I am satisfied with the virtual social networking opportunities I had today.	2 (3%)	5 (7.6%)	13 (19.7%)	20 (30.3%)	26 (39.4%)
	If this event would have been in-person (and COVID was not an issue), would you have attended the event?				
Response	# of Participants		% of Participants		
Yes	48		72.7%		
No	18		27.3%		

We also collected formative feedback to help inform future iterations of the boot camp, at both the in-person and virtual events. While a detailed qualitative analysis was not performed on this data, two authors reviewed all narrative comments and identified recurring themes as described in Table 5.

**Table 5:** Themes From Narrative Feedback

Theme	Supporting Comments
Content	“As a relatively new faculty member, I feel more well-prepared to teach in the hospital now. I hope that there will be more sessions to come that expand on these topics for clinical educators.”
	“I know that it can be challenging to meet everyone where they are at when there is such a large group with varied experiences, but it might be nice to offer one or two sessions that are the next level after foundational concepts as those start to feel repetitive.”
	“I think, in general, people in attendance already have an interest in education and thus have some baseline knowledge/skills around teaching frameworks, feedback. etc. I think I was hoping to hone some of my skills, and some of the sessions felt like reinforcement of concepts I already knew.”
	“I also liked how you let everyone select which breakouts they attended. However, it could have been good to have one additional breakout that was also non-clinically focused, similar to the “small groups” session. As a non-clinician, I did not know where to go for my second breakout as none really benefitted me. But I know the majority are clinical educators, so no biggie; it was still very helpful!”
Duration	“This could have been a day workshop instead of three hours.”
	“I would have enjoyed a bit more time to pick others’ brains about how they operationalize some of the strategies we were discussing.”
	“(I) would have liked more time for discussion in the small groups and examples of good and bad feedback.”
Dynamic Facilitators	“This was a very well-thought-out program with dynamic speakers. The sessions were well timed and provided concrete examples on how to improve my teaching/feedback that I plan to immediately incorporate into my clinic and OR. I also loved interacting with colleagues from different specialties.”
Format	“I was staffing Labor and Delivery yet was able to attend most of the event minus ~45 minutes. If it had been in person, I do not think I would have been able to request time away by the time I found out about the boot camp.”
	“It would be great if next year was semi-virtual where we could gather and see the lectures but be able to break off into face-to-face small groups.”

## Discussion

In this study, we have described an effective faculty development boot camp teaching core competencies for medical clinician-educators. We demonstrated significant improvement in knowledge scores related to session objectives for several sessions in both the in-person and online iterations of the boot camp. Our findings reflect similar results from the systematic review of Steinert et al. [18]. In the 2020 in-person boot camp, there was a significant improvement in knowledge scores for two of the five sessions analyzed (one was excluded due to only one respondent), with nonsignificant trends toward improvement in the remainder. In the 2021 online boot camp, we showed significant improvement in knowledge scores for four of the six sessions with nonsignificant trends toward improvement in the remainder.

Given the similarity of the curricular content, the online format of the 2021 boot camp did not detract from knowledge acquisition and may have been more effective in some instances. However, we suspect that the small sample size was the primary reason for fewer statistically significant improvements in the 2020 boot camp. Additionally, there appears to be a ceiling effect for scores on the evaluation session in both years. This may be due to participants already being familiar with the evaluation tools used at our institution, considering that the average number of years of teaching was seven to eight.

The participants reacted positively to both the in-person and virtual editions of the teaching boot camp, with nearly all reporting that they would recommend it to others for both years. There was a significant difference in the content rating between years, with more participants in 2021 rating the content as “somewhat helpful” than in 2020. This may have been an effect of the larger group or the transition to the online format being less favorable to some participants. While the participants generally found the online platform easy to navigate, they were less satisfied with the networking opportunities on the online platform. Several narrative comments reflected this sentiment as well, noting the benefits of making in-person connections at educational events.

Narrative comments were generally positive as well with participants indicating they found the content applicable to their teaching practice. Multiple comments did indicate a preference for an in-person format, although busy clinicians appreciated the opportunity to attend a high-yield boot camp while multitasking if needed, without needing to take a full day away from clinical duties. Such multitasking may detract from engagement with content, but the demonstrated improvement in knowledge domains supports the effectiveness of the format [19]. Additionally, the participants noted that the in-person boot camp could have easily been a full-day event, whereas online participants cited “Zoom fatigue” after the four-hour event. Narrative comments also drew attention to the value of having an energetic, engaged moderator to help participants transition between sessions during an online event.

Our study has several limitations. First, the response rate to the needs assessment survey was low and likely biased toward faculty who already had a strong interest in clinical teaching. This could have led to the exclusion of content that would have been helpful to cover for less experienced faculty. However, we are confident that our content was relevant given that the participants had on average 7-8 years of teaching experience and still reported finding the content useful. Second, while we were able to measure reaction to the content and immediate knowledge acquisition, we did not capture delayed knowledge retention, changes in teaching practice, or impact on learner outcomes. Future directions of study to address this gap include direct observation and evaluation of faculty teaching, comparison of student evaluations of faculty who have completed the training to departmental averages, and changes in student perception of the clinical learning environment.

## Conclusions

Many medical schools utilize preceptors in distant locations. We demonstrated the feasibility of reaching a much larger and geographically widespread group of clinical preceptors using a virtual format while still showing improvement in knowledge scores relating to workshop content. This was done with a much lower cost as well. Utilizing a virtual platform to deliver faculty development can allow medical schools to create a more uniform and positive learning experience for their students traveling to satellite campuses for their clinical locations. For future faculty development, we anticipate that hybrid models with both in-person and virtual components will be effective in meeting the needs of a geographically distributed faculty. Based on feedback, we would suggest ensuring adequate support for online participants including ensuring that facilitators are present in all breakout rooms and all instructions be provided in advance in case technical issues lead participants to miss content.
